# Unobtrusive, natural support control of an adaptive industrial exoskeleton using force myography

**DOI:** 10.3389/frobt.2022.919370

**Published:** 2022-09-12

**Authors:** Marek Sierotowicz, Donato Brusamento, Benjamin Schirrmeister, Mathilde Connan, Jonas Bornmann, Jose Gonzalez-Vargas, Claudio Castellini

**Affiliations:** ^1^ Institute of Robotics and Mechatronics, German Aerospace Center (DLR), Erlangen, Germany; ^2^ Artificial Intelligence in Biomedical Engineering, Friedrich-Alexander-Universität Erlangen-Nürnberg, Erlangen, Germany; ^3^ Global Research, Ottobock SE and Co. KGaA, Duderstadt, Germany

**Keywords:** force myography, machine learning, adaptive support, exoskeletons, human–machine interaction

## Abstract

Repetitive or tiring tasks and movements during manual work can lead to serious musculoskeletal disorders and, consequently, to monetary damage for both the worker and the employer. Among the most common of these tasks is overhead working while operating a heavy tool, such as drilling, painting, and decorating. In such scenarios, it is desirable to provide adaptive support in order to take some of the load off the shoulder joint as needed. However, even to this day, hardly any viable approaches have been tested, which could enable the user to control such assistive devices naturally and in real time. Here, we present and assess the adaptive Paexo Shoulder exoskeleton, an unobtrusive device explicitly designed for this kind of industrial scenario, which can provide a variable amount of support to the shoulders and arms of a user engaged in overhead work. The adaptive Paexo Shoulder exoskeleton is controlled through machine learning applied to force myography. The controller is able to determine the lifted mass and provide the required support in real time. Twelve subjects joined a user study comparing the Paexo driven through this adaptive control to the Paexo locked in a fixed level of support. The results showed that the machine learning algorithm can successfully adapt the level of assistance to the lifted mass. Specifically, adaptive assistance can sensibly reduce the muscle activity’s sensitivity to the lifted mass, with an observed relative reduction of up to 31% of the muscular activity observed when lifting 2 kg normalized by the baseline when lifting no mass.

## Introduction

An exoskeleton, as commonly defined in robotics, is a mechanism typically consisting of a series of rigid links coupled with the individual segments of the user’s limbs, normally with the aim of increasing strength or facilitating movements (Yang et al., 2008; Anam and Al-Jumaily, 2012). In the industrial setting, exoskeletons can aid workers dealing with tasks which could otherwise lead to serious work-related musculoskeletal disorders (WRMSDs). (Yamamoto et al., 2002; Huysamen et al., 2018). Tasks involving manipulating or holding heavy objects overhead are linked with a variety of WRMSDs in the shoulder (Bjelle et al., 1979), especially when associated with the requirement of keeping the arm at a higher angle from the torso (Svendsen et al., 2004). Exoskeletons could be used to provide support against gravity when this sort of posture cannot be avoided. Examples of such exoskeletons which are currently available on the market include the *ShoulderX* by SuitX (SuitX, 2021), Comau’s *Mate* (Comau, 2021), and the *PAEXO Shoulder Support* by Ottobock (Ottobock, 2021), all of which are designed to provide support at the shoulder joint through a passive spring mechanism. They have been shown to reduce short-term physical strain when performing tasks involving, for instance, holding a heavy tool above the head level or maintaining an awkward pose (Alabdulkarim et al., 2019; Maurice et al., 2019; Schmalz et al., 2019; Nelson et al., 2020; Fritzsche et al., 2021). Although long-term data are not yet available, these devices show promise for reducing health risks for the workers, decreasing their likelihood of incurring into shoulder WRMSDs.

However, in the aforementioned examples, the level of assistance provided by the exoskeleton can only be set manually by changing the spring stiffness parameter or the lever arms. In the literature, certain solutions are presented where the spring offset is set by a motor, but this still needs a manual input by the experimenter or the user (Grazi et al., 2020); this factor usually induces the designers to provide the support mechanism with a lower limit for the maximum force that can be exerted on the user, as the device could otherwise cause difficulties for the wearer when trying to lower their arms from a raised position. An intention-based control system, able to actively set the level of support online without the need for the user to manually input the desired level of assistance, on the other hand, could allow the designers to provide their exoskeletons with higher output torques. In Missiroli et al. (2022), a concept is presented where the level of assistance in a tendon-based system is determined based on the angle of the arm with respect to the body. Although the system presented there did not automatically change the level of assistance provided to the shoulder joint, but rather only the assistive torque exerted on the elbow, keeping the user’s posture into account would definitely enable the controller to adapt the provided assistance in a natural fashion. Here, we present a solution that adapts the level of assistance based on the weight of the lifted object. This estimate is achieved by measuring muscular activity, thus providing an appropriate level of support without the need for conscious participation by the user, thus decreasing the overall mental workload as opposed to a setup where the user has to manually set the level of assistance.

The most traditional means to measure muscle activation, namely, surface electromyography (sEMG, Merletti et al. (2009)) has often been investigated in the literature as a possible mean of controlling exoskeletons (Singh et al., 2012). For example, Gopura et al. (2009) proved the effectiveness of an impedance control–based model using sEMG activity and upper-limb posture for controlling a 7DOF exoskeleton. However, this method is hardly viable in industry, as it would be unpractical to fit a worker with a set of sensors which need to be in direct and constant contact with the skin. In general, there is a lack of robust and accepted ways to let a user control an upper-limb exoskeleton, which would also be practical in an industrial setting. While pursuing this goal, in this work we turn our attention to a cheaper and easier-to-use alternative to sEMG, namely, force myography (FMG, Curcie et al. (2001), Wininger et al. (2008), Ravindra and Castellini, (2014), Radmand et al. (2016), Connan et al. (2016)). This sensor technology relies on measuring muscle bulging upon contraction, usually by means of a force sensor pressed onto the body. FMG sensors do not need to be in direct contact with the skin, and can be easily integrated in a harness worn above the clothing. A further advantage is that implementation of this kind of sensors can be extremely cheap, as force can be measured by means of a simple strain gauge, while still providing measurements so accurate that they can be used in order to control prosthetics (Cho et al., 2016).

Of course, FMG suffers from issues as well. Examples include saturation and bias of the measured signal, as well as problems of cross-talk between muscle groups depending on the harness design, for instance, bulging of one muscle could lead to an increase in pressure on sensors diametrically opposed, if the sensors are arranged in a bracelet. Still, there are already examples in the literature of FMG usage to control exoskeletons. In Islam and Bai, (2019) the authors determine three payload levels through support vector machines with FMG sensors as an input. However, in this case, the exoskeleton was not providing any support and was used passively. Ebrahimi et al. (2017) developed and tested on one participant a method for adjusting the control parameters of their exoskeleton in real time by using several measurements: joint angle, speed, force sensors on the lower and upper arms, and force-sensing gloves. In this case, the control parameters were modified based on a single calibration round, and were not changed in real time. Adopting a slightly different approach, Huang et al. (2015) and Miller and Rosen, (2010) both used force sensors to compute a trajectory that the exoskeleton would help to execute. In these cases, the experimenters used non-movable rehabilitative exoskeletons with active, non-compliant motors assisting each joint of the user.

The aforementioned studies have evaluated the use of FMG for exoskeleton control, but in no case, to the best of our knowledge, the approach has been fully evaluated online. The exoskeleton used is a fully portable solution employing a compliant and lightweight actuation mechanism able to provide an adaptive support via a motor changing the lever arm distance between the arm and the support bars, allowing to change the support torque at the joint.

In order to test the feasibility and the performance of the concept proposed here *in an online setting*, 12 users were recruited to perform a set of repetitive pickup–hold–carry–release tasks, while a regression-based machine learning algorithm used FMG measurements to estimate the weight lifted by the user, and appropriately adjust the level of assistance provided by an industrial exoskeleton in real time. The exoskeleton of choice was an adaptive prototype built by Ottobock, based on a modified version of the Paexo Shoulder Support (Ottobock, 2021). The modified version of the Paexo will henceforth be denoted as *Adaptive Paexo Shoulder*. Because the setup presented here is based on an assistive device which has already been tested and characterized (Maurice et al., 2019; Ottobock, 2021), the focus of this study was not to confirm the effects of exoskeleton’s fixed support as compared to the unassisted condition, but rather as compared to an intent-based adaptive assistance condition. We hypothesized to observe a more constant muscular effort over different lifted masses when using adaptive assistance, as opposed to a fixed passive assistance. In other words, we expected a diminished increase in muscular activity in the shoulder muscles as a consequence of increasing the lifted mass. The results of this evaluation are extremely promising in this sense, showing furthermore that adaptive assistance increases kinematic stability of the shoulder joints enabling more precise movements. The strength of this study resides in demonstrating the feasibility of real-time control on a semi-active exoskeleton using force myography (FMG) as input. FMG sensors are potentially far more practical in real-world applications than their EMG counterparts. Furthermore, to the best of our knowledge, this is the first study showcasing the online control of a supportive exoskeleton based on the estimation of the lifted mass.

## Materials and methods

### The adaptive Paexo Shoulder

**FIGURE 1:**
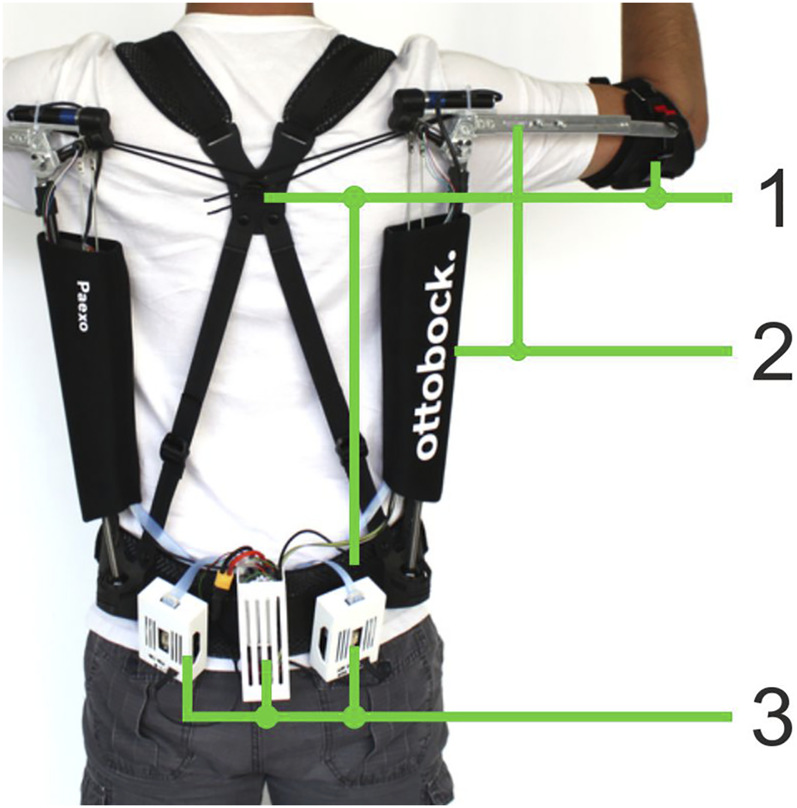
Adaptive Paexo Shoulder as worn by a participant. 1) Textile support structure, 2) assistive structure, and 3) control electronics including power source.

**FIGURE 2:**
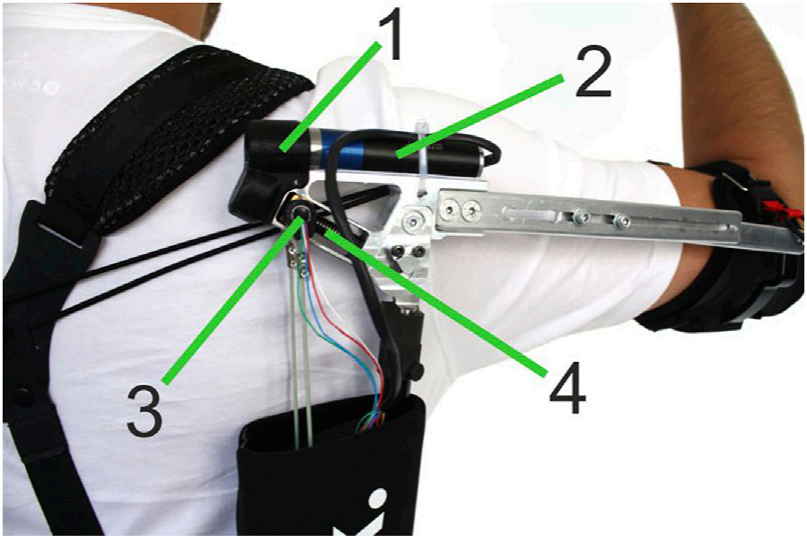
Actuated joint of the adaptive Paexo Shoulder: 1) axe compensation, 2) brushless dc servo motor, 3) nut with encoder, and 4) trapezoid leadscrew.

The adaptive Paexo Shoulder (Figure 1) expands upon the basic Paexo design from Ottobock (2021), as it features the possibility to automatically set the overall support provided by the passive spring-based actuator. For this purpose, a DC brushless Faulhaber 2057B motor is integrated directly at the shoulder joint (Figure 2). The motor can be used to change the length of the lever arm with which the spring element pulls the humeral orthosis. Therefore, the adaptive Paexo Shoulder still behaves like a passive device, but allows to automatically change the operating point of the of the spring mechanism, effectively increasing or decreasing the overall support provided to the user. This mechanism introduces a certain latency in the control loop, as a transition from the minimum to the maximum lever arm can last up to 2 s. However, internal testing shows that, because the system is always providing some level of support, this latency has no issue and the user still perceives the support as transparent. In the setup presented here, the level of support depends on various anthropometric measurements of the user, according to Eqs 3–6. The adaptive Paexo Shoulder has the same frame and structure characteristics as the Paexo: the actuator is mounted on the arm bar, which is connected to the support bar via an expander rope. This acts as a spring that generates a torque in the joint as a function of the arm anteversion. A textile stabilization harness supports donning and doffing, and keeps the structure close to the torso. The only semi-rigid structures coming into contact with the body are the belt and the two underarm cuffs. A rotational encoder is used to measure the joint angle. The entire setup weighs 3 kg and can be used with a 14.8V/1550mAh LiPo battery for 6–8 h, depending on the amount of usage.

### Force myography bracelets

**FIGURE 3:**
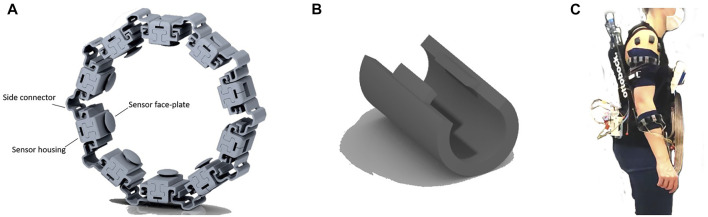
CAD view of the bracelets and bracelet elements. **(A)** Render of an assembled FMG bracelet with 10 sensing elements. **(B)** Render of one rigid clip which can be used to clamp the lateral connectors to fit the bracelet to limbs of lower circumference. **(C)** Full FMG setup as worn by a participant during the experiment. Notice also the EMG sensor probes worn on the shoulder.

In the presented setup, the required support level of the adaptive Paexo Shoulder was calculated based on measurements from a set of FMG sensors. As FMG entails measuring the force from muscle bulging, the design of the harness pressing the sensors onto the body segments to be monitored is paramount. The FMG sensors were lodged in individual housings, which were in turn arranged in two modular bracelets worn by the user on the forearm and upper-arm, respectively. The FMG sensors (*FSR 400 short* by Interlink Electronics, (Electronics, 2022)) are integrated in an analog amplification circuit designed internally (see (Connan et al., 2016)). The amplification circuit board and the FSR were housed in flexible 3D-printed housings, as shown in Figure 3. The armbands (as shown in Figure 3) consisted of four elements:

• Sensor housing main body, which holds the sensor and binds the shell assembly to the lateral connectors.
• Sensor face-plate, which conveys and directs the pressure from muscle bulging directly on the sensor strain gauge. The two sub-elements are connected by means of snap-on appendages on the face-plate.
• Side connectors, designed to be easily extendable, thus providing flexibility over a range of arm sizes, with the possibility of being fitted with a rigid clip, which is used to ensure that the bracelet fit limbs with smaller circumference.
• Binding clips, whose purpose is constraining the length of connectors as described earlier to fit limbs of smaller circumference.

Because of the modular design, the number of sensors for each bracelet can be changed, and fine adjustments can be made as required in order to improve the fit on any individual user, either by using the binding clips, or by adding additional connectors and shells. The bracelet was manufactured via fused deposition modeling out of TPU material, with shore hardness 90A, which gives the outer surfaces a gritty texture making the friction between connectors and shell bodies such that no additional fasteners are required to keep the elements in place. The used force-sensing resistors are shown in Castellini and Ravindra (2014) and Connan et al. (2016) to have an extended linear region in the sensitivity curve. The bracelets were designed and tested to keep the sensor in this linear region.

**FIGURE 4:**
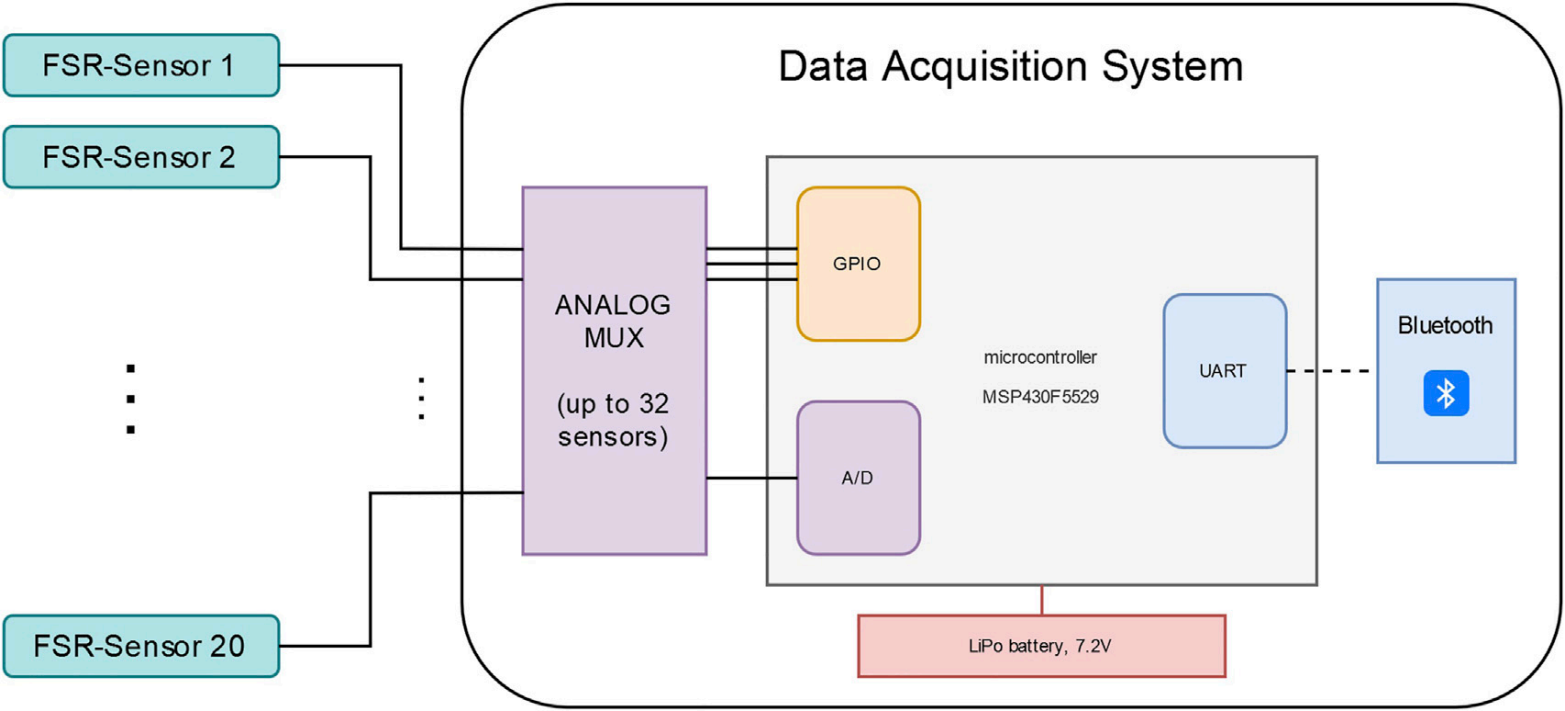
DAQ system for FMG: overview of the major components.

The measurements from the FMG sensors were acquired and transmitted to a remote host via a data acquisition (DAQ) system shown in Figure 4. The DAQ board used here is an updated version of the one presented in Connan et al. (2016).

**FIGURE 5:**
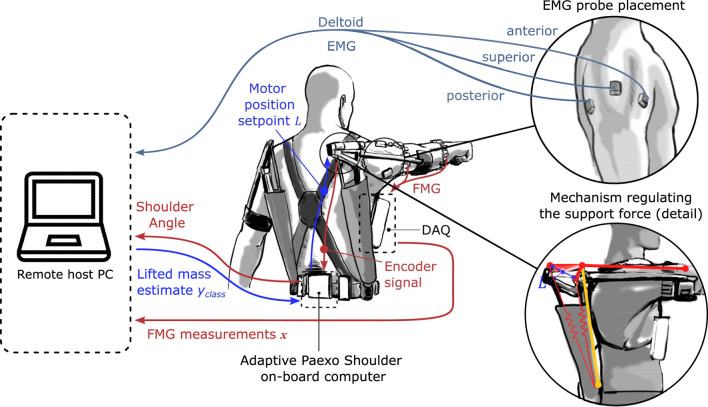
Block diagram of the control loop regulating the level of assistance and detail of EMG sensor placement.

The DAQ board is designed around a low-power microcontroller (MSP430F5529, by Texas Instruments) running at 25 MHz. During normal operation, the system draws approximately 80 mA, which translates to more than a day of continuous operation when powered by a 7.2 V 2400 mAh LiPo battery. The DAQ can acquire data on up to 32 hard-wired channels. The analog signal from these channels is converted to digital with 12 Bits resolution. In the setup presented here, only 20 FMG sensors are used. In order to facilitate the integration of remote hosts in the control loop, the adaptive Paexo Shoulder can be controlled through a wireless interface over a Bluetooth module (RN42 from Roving Networks). In the setup presented here, a remote host PC ran the prediction algorithm which estimated the mass lifted by the user (see *Estimation of the required support*) and translated it to a desired support level. The communication between the host computer and the adaptive Paexo Shoulder controller was based on a serial protocol and the out- and inbound messages were checked at a rate of 200 Hz. The control loop and the sensors used to monitor the muscular effort during the study are shown in Figure 5.

### Estimation of the required support

To estimate the support desired by the participant in real time starting from the 20 FMG signals, we used standard ridge regression (Hoerl and Kennard, 1970). Let $x\in R^{20}$ denote the signal vector; then the output of the ridge regression estimator is *y* = ***w*** ^*T*^ ***x***, where $w\in R^{20}$ is obtained through regularized minimization of the mean-squared error, leading to the following closed form solution for ***w***

$$w={\left(X^{T}X+\lambda I\right)}^{-1}X^{T}y,$$ (1)

where $X\in R^{20\times N}$ is the design matrix gathering *N* observed sensor measurements of the form ***x***, and $y\in R^{N}$ is a vector gathering the weight lifted in association with each observation ***x*** present in *X*. Although the weights in this vector were not in kilograms, the values were proportional and subsequently discretized and scaled as shown in Eq 2. *X* and ***y*** must be collected at the beginning of each experiment in order to create an appropriate training set for the calibration of the ridge regression model and provide a sensible estimation of the optimal ***w***. *λ* represents a regularization term, which keeps the parameters in ***w*** low in magnitude.

The FMG signals were sampled by the DAQ board at 192.5 Hz, and then wirelessly transmitted to a host computer where they were filtered with a first order Butterworth filter, with cut-off frequency of 1 Hz to extract slower dynamics. For the calibration procedure, the filtered signals were then fed to the ridge regression algorithm with *λ* = 1, which was trained once at the beginning of each experimental round (i.e. only once per subject), only allowing for initial re-calibrations if the prediction was visibly unstable. The reasons for instability of the prediction are most likely wrong sensor placement or an erroneous performance of the actions required for the calibration. During the calibration procedure, the FMG signals would be sampled for 10 s while the participant performed one of the following actions per sampling:

• Both arms relaxed and kept along the sides, hands unclenched.
• Right arm raised at 45° over the horizontal plane, no weight held.
• Right arm raised at 45° over the horizontal plane, holding a 1 kg weight.
• Right arm raised at 45° over the horizontal plane, holding a 2 kg weight.

These correspond, in turn, to the following labels: *y*_*l*,*i*_ ∈ [0, 0.1, 0.5, 1.2]. These values for the response variable were chosen based on previous tests. The dataset consisted, therefore, of approximately 1925 samples for each of the four labels. The training of the regression model takes under 1 s with this number of observations. The time efficiency in the training phase is the main reason why a ridge regression model was chosen for this application. Although no circumstances were observed during the course of the experiment where a model recalibration was necessary, the short duration of the procedure would make it possible to easily recalibrate the model, should the sensors need to be repositioned or re-instrumented ina real-world scenario. After training, the regression algorithm provided a 1-dimensional estimation *y*_*pred*_ of the user’s effort based on signals filtered analogously to those used for calibration. The obtained prediction was then additionally low-pass filtered, clipped between 0 and 1, and subsequently discretized in three levels, according to the following criteria:

$$y_{\mathrm{class}}=\left\{\begin{array}{cc} 0\; & \;\text{if }0\leq y_{\mathrm{pred}}<0.35\\ 1\; & \;\text{if }0.35\leq y_{\mathrm{pred}}<0.75\\ 2\; & \;\text{if }y_{\mathrm{pred}}\geq 0.75 \end{array}\right.,$$ (2)

The prediction step of the regression model is instantaneous when compared to the latency due to the adaptive Paexo Shoulder’s mechanism. The value of *y*_*class*_ was communicated to the adaptive Paexo Shoulder (Figure 5) in order to issue support levels for, respectively, 0 kg, 1 kg, or 2 kg weights. The exact amount of force provided to the user was computed on the adaptive Paexo Shoulder’s internal controller, depending on each user’s biometrics (specifically body weight and height, using the relations shown in Eqs 3–6). Although the FMG sensors used in the experiment have non-negligible hysteresis at high forces, as well as a non-linear transfer function, it was shown in Ravindra and Castellini (2014) that for moderately high forces (0–15N), like those that could be produced by muscle bulging, the behavior is fairly consistent and their transfer function is nearly linear. For this reason, it was not deemed necessary to make use of more advanced ML algorithms to account for non-linearity.

The conversion from the algorithm’s estimate to lever arm length takes into account the estimated lifted weight *y* as well as the user’s body mass *m* and arm’s length *l* according to the following laws:

$$m_{\mathrm{lifted}}=m\left(f_{ua}+f_{fa}+f_{h}\right)+y,$$ (3)

$$h_{\mathrm{COM}}=\frac{mf_{ua}h_{ua}+mf_{uf}h_{fa}+\left(mf_{h}+y\right)h_{h}}{m_{\mathrm{lifted}}},$$ (4)

$$\tau =0.7gmh_{\mathrm{COM}},$$ (5)

L=0.05523τ+3.007, (6)

where *f*_*ua*_, *f*_*fa*_, and *f*_*h*_ represent the percentage of the bodyweight constituted by the upper-arm, forearm, and hand, respectively, *h*_*ua*_, *h*_*fa*_, and *h*_*h*_ represent the position of the centers of mass for the upper-arm, forearm, and hand, respectively, and *g* is the gravitational acceleration. All of the remaining constants were empirically determined during pre-tests in order to provide a good level of support without exerting too much pressure on the user. The bodyweight coefficients and the positions of the centers of mass are all based on anthropometric tables found in Drillis et al. (1964). The positions of the individual centers of mass are based on the length of the user’s arm with the arm in a standard working position, which in this case was assumed to be constant across all tasks. Therefore, after the initial calibration and during the session, the level of assistance only changes as a function of the estimated lifted mass. The actual support force provided during the experiment depends on the characteristics of the user and on the shoulder anteversion angle, but for reference, the Paexo can provide a maximum support force of around 50 N.

### Participants

Twelve participants (nine males, three females, 27.6 ± 2.9 years old, 71.9 ± 6.5 kg, 1.76 ± 0.07 m) were involved in a repetitive series of tasks, designed in order to require different levels of assistance at different times. The study design was within-subject: the participants were divided in two subgroups. Group A performed the tasks with the adaptive assistance on first, and then performed them again with the adaptive assistance off and the Paexo set to a mid-scale support force. Group B completed the tasks with the conditions inverted. This subdivision had the goal of counterbalancing the effects of fatigue over time on the outcome metrics. All users were thoroughly informed about the experiment before taking part in it, both orally and in writing, and then signed an informed consent form. The experiment was carried out in conformity with the WHO Helsinki Declaration and was authorized by the DLR internal committee for safety and data protection. Although the end-user group would presumably also consist of able-bodied individuals, no particular effort was put in assuring that the study population would match the end-user group in terms of age, gender, or BMI distribution.

### Experimental setup

For the purposes of the experiment, in addition to the FMG sensor setup and the on-board sensors of the adaptive Paexo Shoulder described earlier, the participants were fitted with three *Trigno* EMG sensors by DelSys. These were placed on the anterior, superior, and posterior deltoid of the right arm, respectively, as shown in Figure 5. Although dorsal muscles are also involved in the overhead work, the EMG probes were placed exclusively on the deltoid muscles because the results presented in Maurice et al. (2019) suggested that the support force provided by the Paexo has the most significant effect on the activity of this muscle group, and does not significantly affect the activity of dorsal muscles. By extension, no significant differences could realistically be expected on dorsal muscle activity when comparing passive and adaptive assistance. The EMG measurements were bandpass filtered between 20 and 450 Hz. The feed from the sensors was sent to the host PC at a rate of 2000 Hz. For the purposes of the offline analysis, the absolute value of the EMG was extracted. The average and standard deviation of the EMG were extracted over the whole time during which the task was computed. The maximal value of the EMG measured for each participant was used as a normalizing factor.

The EMG measurements were used, among other things, to compute the muscular effort ratio *r*_*d*_, defined as the ratio of the mean absolute value of the EMG on the deltoids (the index *d* indicates the deltoid group) when lifting 2 kg and when lifting 0 kg, in accordance with the following relation

$$r_{d}=\frac{\bar{\left|EMG_{m=2\;\text{kg}}\right|}}{\bar{\left|EMG_{m=\text{0}\;\text{kg}}\right|}}\text{, }d\in \left\{\text{ant., sup.}\right\}.$$ (7)

This is indicative of the rate at which muscle activity increases as a consequence of increasing lifted mass. What we set out to demonstrate is that the adaptive assistance significantly decreases this rate when compared to the non-adaptive assistance.

**FIGURE 6:**
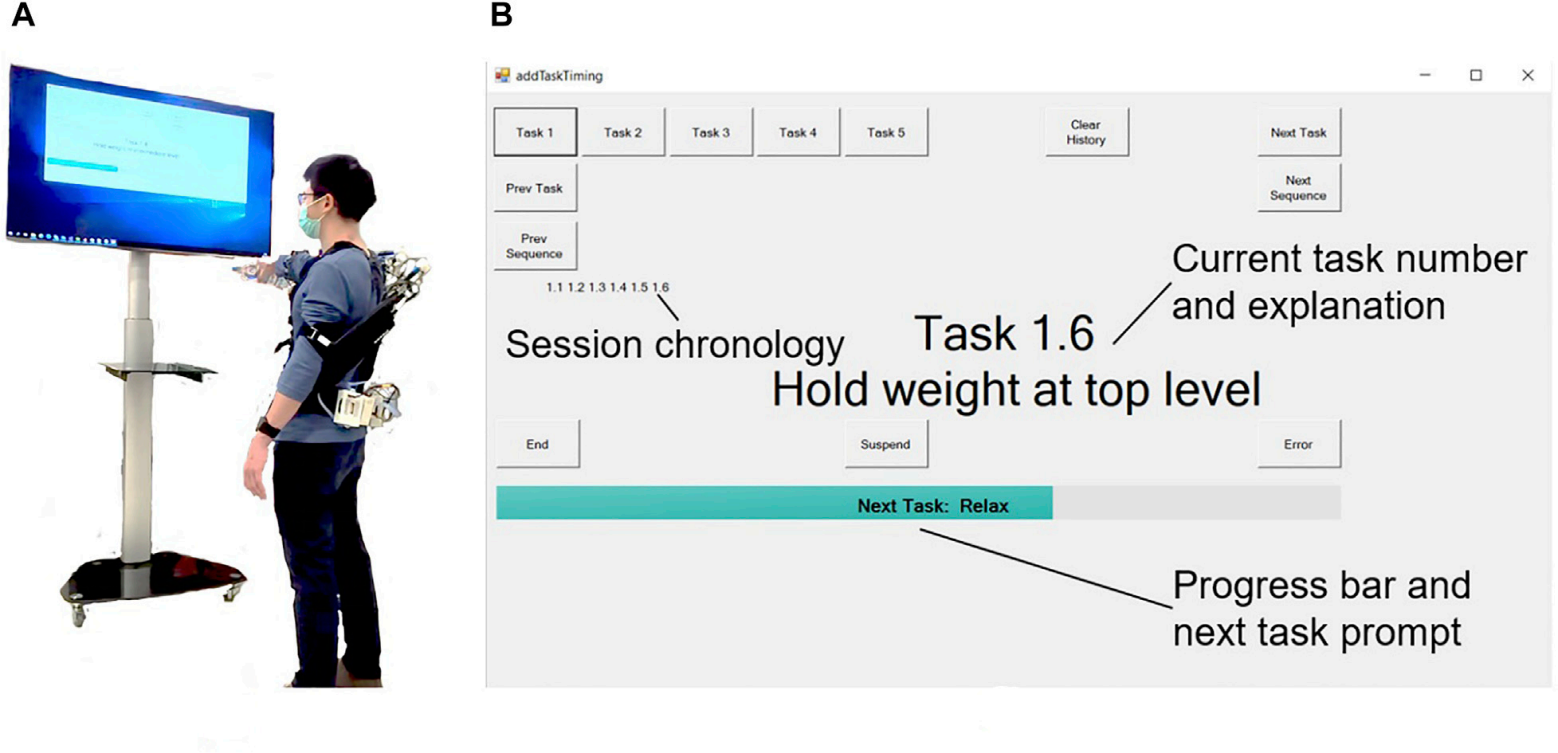
Experimental setup. **(A)** Participant performing the experiment. **(B)** GUI.

The participants were asked to maintain their shoulders parallel to a screen placed in front of them, which showed them a GUI guiding them through the sequences by showing prompts and the remaining duration of each task. On the participant’s right, a stack of shelves served to store the weights used throughout the session. Markings were drawn on the shelf in order to help the participants find the reference points for the two main angles at which they were required to hold their right arm. The experimental setup and GUI are shown in Figure 6. The stability of the shoulder angle as measured by the exoskeleton’s internal encoder was also used as an evaluation metric. Specifically, the standard deviation on the shoulder angle is measured by the Paexo’s on board shoulder encoder. This is indicative of the precision with which the user is able to maintain a position or a trajectory.

### Experimental protocol

**FIGURE 7:**
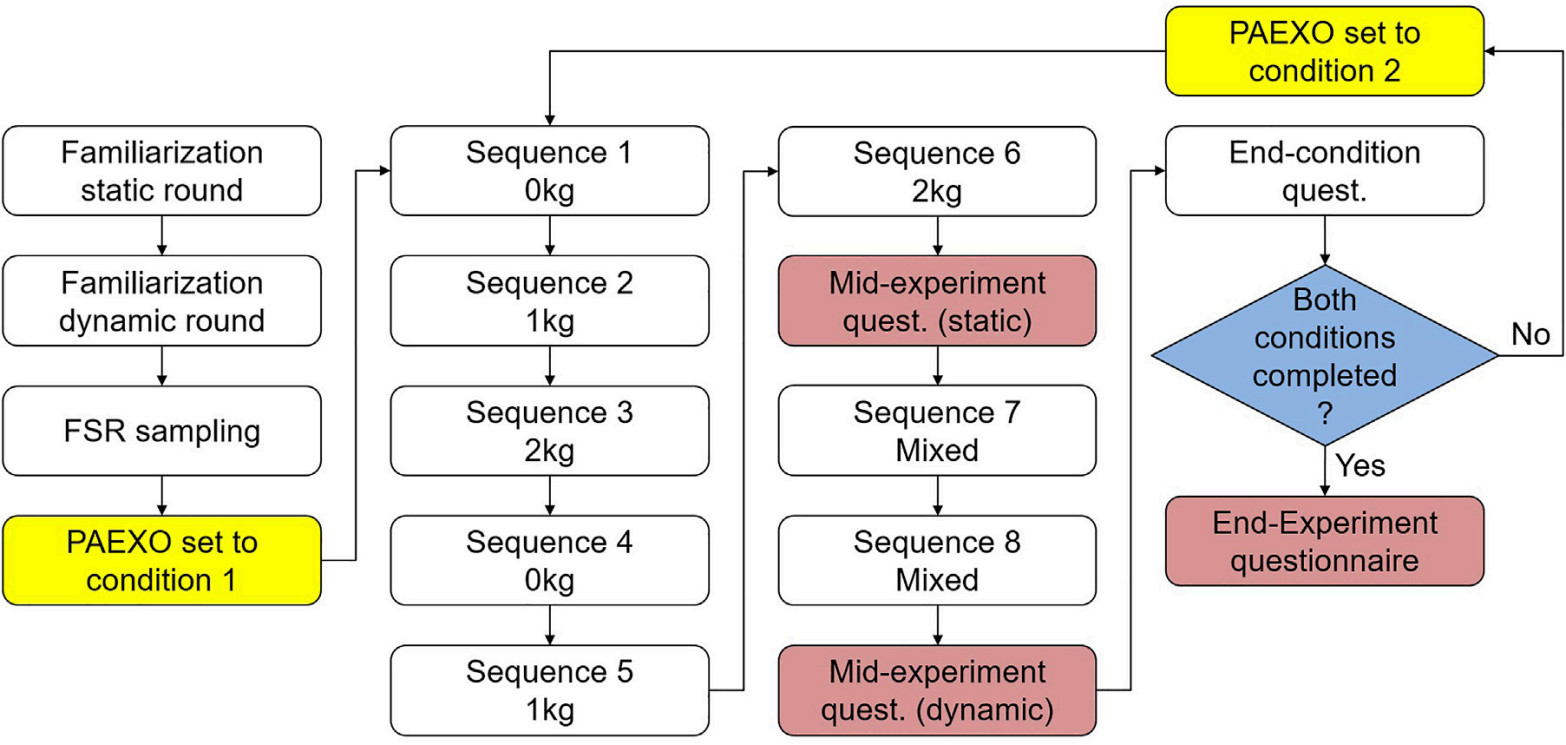
Flow-chart of the experimental protocol.

**FIGURE 8:**
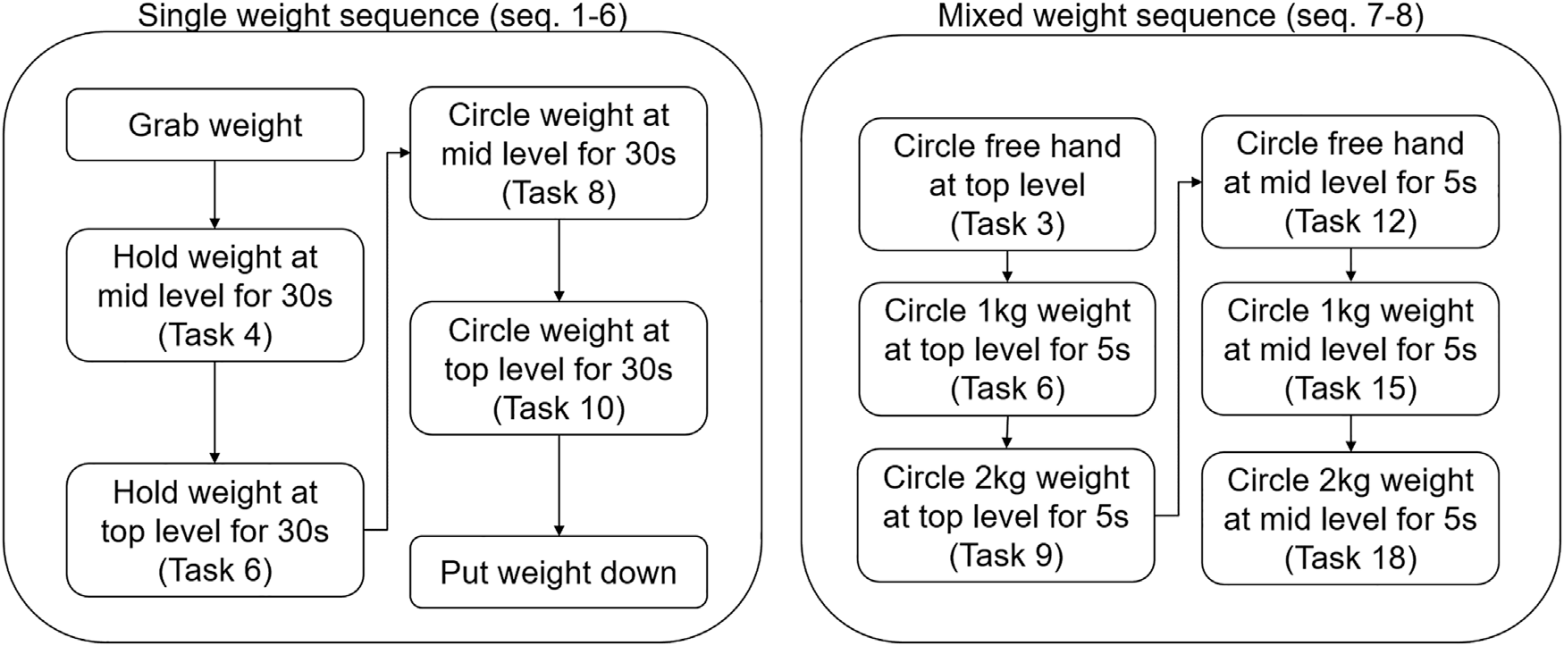
Detailed breakdown of each sequence.

After providing their informed consent and general data, the participants were fitted with the exoskeleton and the sensor setup. The sensor density was sufficient to ensure that muscle activity caused by lifting a mass would be measurable independent of the bracelet’s orientation around the body segment’s longitudinal axis. The two bracelets were positioned in order to cover the region of largest diameter on both the right forearm and the right humerus, and the positioning of the sensors was never changed throughout the experimental session. After being fitted with the devices and after performing a familiarization round, the participants performed the calibration procedure described in the *estimation of the required support*. During the session proper, the participants were asked to perform a series of tasks involving holding either 0 kg, 1 kg, or 2 kg with the arm horizontal or at about 45° above the horizontal plane for 30 s. The weight was to be held either in an isometric contraction or moved in circles counter-clockwise at about one round per second. All of these tasks were to be repeated twice. The combination of these factors gives sequences from 1 to 6 as shown in Figure 7. The relevant tasks for this sequence are shown on the left side of Figure 8. After completing these, the participants were asked to compile a mid-experiment questionnaire specific to these sequences. The participants were then asked to perform two further sequences. These required the participants to pick 0 kg, 1 kg, or 2 kg from a lower shelf, move them in circles close to a higher shelf, and then leave them there, and then repeat these tasks starting with the higher shelf. This sequence of tasks was to be repeated twice, and these two repetitions are shown as sequences seven and eight in Figure 7. The relevant tasks for these sequences are shown on the right side of Figure 8. The order in which the tasks were performed was not randomized, as the goal of the study was not to ascertain an effect of the task on the muscular activity, but only the effects of the adaptive assistance as opposed to the non-adaptive assistance. After this, the participants were asked to fill a mid-experiment questionnaire about sequences seven and eight, as well as a condition-specific mid-experiment questionnaire.

All participants completed all tasks and sequences with both adaptive and non-adaptive assistance. As the expected effect of the adaptive assistance was not to decrease the measured muscular activity overall, but rather to decrease the overall sensitivity of the muscular activity to the lifted mass, the level of support force set for the Paexo under the non-adaptive assistance condition is irrelevant. During the experiment, the Paexo was set to a mid-scale support force in order to drive the system at its average operating point. At the end of the session, the participants were asked to compile a post-experiment questionnaire. Here, as well as in the mid-experiment questionnaires, the participants were asked to assign a score from 0 to 20 to a set of task load metrics, in accordance with the NASA Task Load Index assessment Hart and Staveland, (1988). Furthermore, in the post-experiment questionnaire, the subjects were asked to assess the modified version of the adaptive Paexo Shoulder they used with a reduced version of the System Usability Score test (SUS, Brooke. (1996)). The SUS consists of a series of questions to be answered on a 5-level Likert scale. The questions are formulated in such a way that, when evaluating a maximally usable system, the answers should ideally alternate between the maximum and the minimum value on the Likert scale. This is to avoid response repetition bias. The particulars of the two experimental conditions were not explained to the participants. In spite of this, it was not possible to carry out the experiment with the participants completely blind to the current condition, as the adaptive assistance causes the Paexo’s Shoulder motor to move and emit audible sounds, and moreover, one can easily detect changes in the level of assistance. This factor could influence the subjective evaluations, but it should not affect the other metrics. The alternating of groups starting with the non-adaptive and adaptive assistance should aid in counteracting possible biases in the subjective assessments.

## Results

**FIGURE 9:**
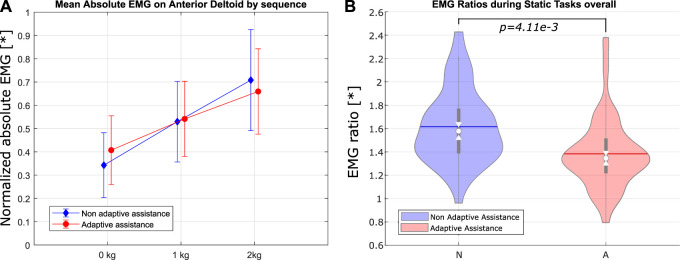
sEMG for single-weight sequences sorted by the assistance mode for the anterior deltoid. **(A)** Bar graph of sEMG measurements by lifted weight. **(B)** Violin plots of the sEMG ratios (sEMG when lifting 2 kg over sEMG when lifting no weight) by the assistance mode.

**FIGURE 10:**
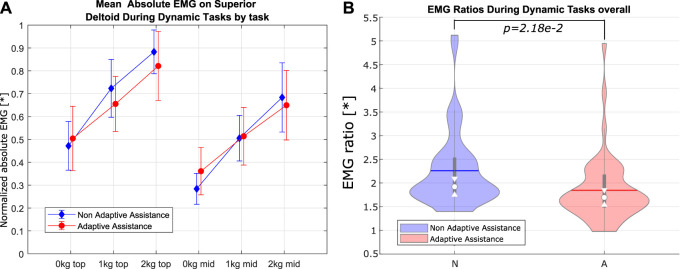
sEMG for mixed-weight sequences, color-coded by assistance mode for the superior deltoid. **(A)** Bar graph of sEMG measurements by lifted weight. **(B)** Violin plots of the sEMG ratios (sEMG when lifting 2 kg over sEMG when lifting no weight) by the assistance mode.

In order to analyze the effects of the different assistance types on the adopted metrics, a repeated measure analysis of variance (rmANOVA) on a multivariate model fitted to the data was performed. The model had the *mode of assistance* as the main independent variable, with value either adaptive or non-adaptive. Within the model, the variables *sequence*, *task*, and *mode of assistance* were all considered within-subject predictors, as all subjects completed all the tasks and sequences with both conditions. The rmANOVA analysis was performed using the Statistics and Machine Learning Toolbox within the Matlab environment (Matlab 2021a; MathWorks, Natick, Massachusetts MA, United States United States) (MathWorks, 2021). The most significant results in terms of *p*-value are listed and explained in the discussion. All main results are reported in Table 1. The assessed metrics are indicated in the leftmost column. The average EMG on the most affected muscle groups and a comparison of the ratios *r*_*d*_ are shown in Figures 9, 10.

**TABLE 1:** Overview of the results for all experimental conditions and significant ANOVA effects were applicable. The metric values are indicated in format *mean (standard deviation)*.

Metric (condition) [Unit]	Non-adaptive assistance	Adaptive assistance	Significant ANOVA or t-test effects of assistance mode
*r*_*ant*_ (Single-weight tasks) [ ]	2.23 (0.69)	1.70 (0.41)	F (1, 11) = 13.02; p < 0.005
*r*_*ant*_ (Task 4) [ ]	2.34 (0.82)	1.86 (0.44)	F (1, 11) = 4.88; p < 0.05
*r*_*ant*_ (Task 6) [ ]	2.17 (0.56)	1.67 (0.28)	F (1, 11) = 16.19; p < 0.005
*r*_*ant*_ (Task 8) [ ]	2.25 (0.77)	1.78 (0.48)	F (1, 11) = 7.17; p < 0.05
*r*_*ant*_ (Task 10) [ ]	2.15 (0.61)	1.49 (0.36)	F (1, 11) = 18.39; p < 0.005
*r*_sup_ (Mixed-weights tasks)[ ]	2.26 (0.80)	1.85 (0.71)	F (1, 11) = 7.12; p < .05
SD on shoulder angle (single-weight seq.) [rad]	0.36 (0.12)	0.33 (0.13)	F (1, 11) = 50.88; p < .001
Subjective assessment of strain (single-weight sequences) [ ]	13.50 (3.40)	11.75 (3.36)	Paired T-test: p < 0.1
Weight estimation error (single-weight sequences) [%]	Does not apply	21.33 (24.72)	Does not apply
Weight estimation error (mixed-weight sequences) [%]	Does not apply	29.91 (32.02)	Does not apply
System usability scale assessment score [%]	Does not apply	74.23 (14.05)	Does not apply

**FIGURE 11:**
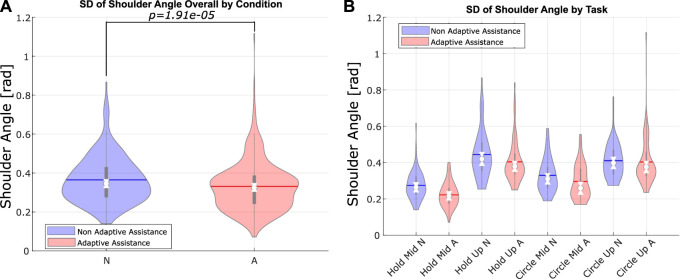
Angular encoder at the shoulder level **(A)** Violin plot of shoulder angle standard deviation by the assistance mode. **(B)** Violin plot of shoulder angle standard deviation by task.

Table 1 also contains the result of a *post hoc* multiple comparison of estimated marginal means, which shows some of the relevant effects sorted by task. As the independent variable, which was the mode of assistance, only has two possible values, no adjustment was needed for the *post hoc* analysis. The subjective assessments (except the SUS) were evaluated on a discrete scale with 20 bins, where the participants had to express the perceived answer to a given question item, based on the NASA Task Load Index (TLX) test (Hart and Staveland, 1988). Figure 11 shows the measured standard deviation of the shoulder angle as measured by the Paexo’s internal encoder. Finally, Table 1 reports the relative error in the estimation of the lifted weight, normalized by the maximum possible error value.

## Discussion

The results presented earlier enable us to characterize many aspects of the presented setup, as well as to draw a few tentative conclusions on the overall effectiveness of the presented setup. Although many solutions involving passive or semi-passive exoskeletons have been presented in the literature in the past (Grazi et al., 2020; SuitX, 2021; Missiroli et al., 2022), intention-based control concepts for such systems are rarely investigated. The main hypothesis of this study is that an intention-based support level selector, used in real time, can effectively reduce the correlation of muscular effort to lifted mass by adjusting the torque and therefore the forces acting onto the user. This would have many potential advantages, and it minimizes the undesired interaction forces between the exoskeleton and the user, which is in general desirable (Ajayi et al., 2020). As the basis of the presented setup is a passive assistive exoskeleton whose main characteristics have been presented in the past (Maurice et al., 2019; Ottobock, 2021), this study does not focus on confirming the effects of the passive support as compared to the case unassisted condition, but rather focuses on identifying the effects of adaptive assistance as compared to a non-adaptive, mid-scale level of support provided by the Paexo.

### Shoulder stability

The standard deviation on the shoulder angle as measured through the Paexo’s encoder is significantly affected by the assistance modality, both in the tasks requiring the participant to hold the weight, as well as in those requiring circular movements. This effect was only detected in the sequences with single weight, most likely because the mixed-weight sequences involved faster movements over shorter times. This would reduce the effect of assistance mode on the shoulder angle stability. Conversely, in the case of single-weight sequences, the higher standard deviation detected under non-adaptive assistance seems to be due to the fact that the participants would slowly lower their arms because of fatigue over the required 30 s of contraction. Figure 11 shows violin plots of this metric overall as well as a breakdown of the tasks.

### Subjective assessment

No effect of the assistance modality on the subjectively assessed metrics can be detected, with the remarkable exception of the *perceived strain* for the sequences involving one weight, which is positively affected by the adaptive modality (*p* < 0.1, Table 1). In the SUS test, the participants evaluated the system with an average score of 74.23 ± 14.05% (Table 1), which corresponds to a B according to the SUS score-grade curve.

### Muscle activity

No significant effect of the mode of assistance on the mean EMG could be determined, when considering all tasks and all sequences. This is likely due to the fact that the adaptive Paexo Shoulder, when in the non-adaptive mode, was providing a mid-scale level of support, which is overall similar to the average level of support provided in the adaptive mode. However, the effect of adaptive assistance, as used within this user study, is statistically significant when one considers the difference in mean EMG activation when lifting no weight and when lifting 2 kg. In particular, the assistance mode shows a significant effect on the ratio between the mean EMG activity when lifting 2 kg and when lifting no weight (the ratios are shown in Figures 9, 10). The effects are most significant for the sEMG on the anterior deltoid during single-weight sequences and for the superior deltoid during mixed-weight sequences (Table 1). A possible reason for this is that the mixed-weight sequences entailed leaving and picking up weights from the shelves on the participant’s right hand side. This would require the user to perform frequent horizontal arm abductions, in addition to the arm flexions and extensions needed in order to lift the weights and to return to the neutral position, which can be largely performed by the anterior deltoid. This could lead to an overall more noticeable recruitment of the superior deltoid, which is mainly used in horizontal arm abduction.

As stated earlier, this indicates that under adaptive assistance, the average muscle activity at the shoulder level does not increase as much when the mass to be lifted increases. If this trend were confirmed over a wider range of support forces, this would indicate that this type of adaptive control *can effectively scale the level of support as the weight to be lifted increases*. As the available support levels of the adaptive Paexo Shoulder increase with the future versions, the adaptive control could likely be used to reduce the amount of muscle fatigue even for higher weights, thereby easing the workload on the user, without increasing the amount of force necessary to lower the user’s upper limbs. Actually, if the exoskeleton were able to provide higher support forces, conceivably even to such a degree that it would be difficult for the user to lower their arms without intention-based control, the adaptive assistance system would likely be able to further flatten the relation between mean sEMG and lifted mass, as shown in Figures 9, 10. There is an assumption underlying this claim, namely, that the adaptive assistance algorithm would then be able to decrease the provided support when needed. This has partially been shown by this study. An interesting fact is that the effect of assistance mode on the EMG ratio is more pronounced, with a *p* value of 1.105*e* − 3, when taking into consideration the second repetition performed by the participants, as opposed to the first one, where the effect has a *p* value of 0.022. This could indicate that the adaptive assistance leads to a slower onset of fatigue compared with the non-adaptive assistance.

### Prediction accuracy

The muscle activity sensing system shown here and the associated prediction algorithm were accurate enough for practical uses. Remarkably, the user-exoskeleton system constitutes a closed-loop system, as the desired support is issued by the adaptive Paexo Shoulder, the muscle activity of the user naturally reduces, thereby reducing the amount of support provided. Evidently, each time the weight to be lifted changes, the user and the exoskeleton reach a new point of dynamical equilibrium in the provided support, balancing each other. In this work we have not explored this relationship, but it is a fascinating research issue and will be investigated in the future. We are especially interested in how modeling this relationship might render the device more ergonomic.

### Study limitations

As is obviously the case, this study has limitations. First, the participants were instructed to only use one particular type of grasp, namely power grasping. Although many studies in prosthetics have shown that machine learning and FMG can easily be used to detect the intent of the users more precisely, the performance is naturally bound to change when allowing for different grasps. Second, we could not use any motion tracking system to determine the potential differences in the motor strategies of the participants, introduced by the adaptive support control. Third, the current range of support that the adaptive Paexo Shoulder is able to provide is rather limited and constitutes a simple case study, which needs to be broadened. These are some aspects which should be addressed by future research. The main goal of future work on this sort of device should focus on generalizing the estimation of the lifted mass to various kinds of grasp, and on the inclusion of posture data in the estimation of the needed assistance, as shown in Missiroli et al. (2022).

## Conclusion

The Paexo has been conceived since its early design stage with non-obtrusiveness and simplicity in mind: it can be donned and doffed easily and quickly and guarantees the full range of motion of the user’s shoulders while worn. The adaptive Paexo Shoulder follows the same design philosophy, and additionally provides adaptive support via a lightweight servo motor. Still, the question remains: *how* to let the user control it transparently, effectively, and in real time? Taking inspiration from the previous work in the field of upper-limb prosthetics, in this work, we have assessed the effectiveness of FMG to determine in real time the amount of support required by the user depending on the lifted mass, and consequently, to control the motor of the adaptive Paexo Shoulder, thereby determining the effects of an adaptive support offered by the device.

A substantial advantage provided by FMG is that it can be worn on the worker’s clothing, as opposed to sEMG sensors, which is an unavoidable constraint in most industrial and commercial settings. Future work will also investigate the integration of further sensor modalities enabling the support force estimator to take into account also the user’s posture, in addition to the estimation of the lifted mass.

## Data Availability

The raw data supporting the conclusion of this article will be made available by the authors, without undue reservation.
